# Caspase-Cleaved Tau Co-Localizes with Early Tangle Markers in the Human Vascular Dementia Brain

**DOI:** 10.1371/journal.pone.0132637

**Published:** 2015-07-10

**Authors:** Ryan J. Day, Maria J. Mason, Chloe Thomas, Wayne W. Poon, Troy T. Rohn

**Affiliations:** 1 Department of Biological Sciences, Science Building, Room 228, Boise State University, Boise, Idaho, 83725, United States of America; 2 Institute for Memory Impairments and Neurological Disorders, University of California Irvine, Irvine, California, 92697, United States of America; McGill University Department of Neurology and Neurosurgery, CANADA

## Abstract

Vascular dementia (VaD) is the second most common form of dementia in the United States and is characterized as a cerebral vessel vascular disease that leads to ischemic episodes. Whereas the relationship between caspase-cleaved tau and neurofibrillary tangles (NFTs) in Alzheimer’s disease (AD) has been previously described, whether caspase activation and cleavage of tau occurs in VaD is presently unknown. To investigate a potential role for caspase-cleaved tau in VaD, we analyzed seven confirmed cases of VaD by immunohistochemistry utilizing a well-characterized antibody that specifically detects caspase-cleaved tau truncated at Asp^421^. Application of this antibody (TauC3) revealed consistent labeling within NFTs, dystrophic neurites within plaque-rich regions and corpora amylacea (CA) in the human VaD brain. Labeling of CA by the TauC3 antibody was widespread throughout the hippocampus proper, was significantly higher compared to age matched controls, and co-localized with ubiquitin. Staining of the TauC3 antibody co-localized with MC-1, AT8, and PHF-1 within NFTs. Quantitative analysis indicated that roughly 90% of PHF-1-labeled NFTs contained caspase-cleaved tau. In addition, we documented the presence of active caspase-3 within plaques, blood vessels and pretangle neurons that co-localized with TauC3. Collectively, these data support a role for the activation of caspase-3 and proteolytic cleavage of TauC3 in VaD providing further support for the involvement of this family of proteases in NFT pathology.

## Introduction

Vascular dementia (VaD) is the second leading cause of dementia in the USA, only trailing Alzheimer’s disease (AD) and accounting for 15–20 percent of all types of dementia [1]. It has been estimated that 25–80% of all dementia cases show mixed pathologies between VaD and AD, therefore, contributing to the difficulty in diagnosing pure VaD [2]. An additional confounding factor in diagnosing VaD is the lack of widely accepted neuropathological criteria for VaD [3]. VaD is classified as a cerebral vessel vascular disease characterized by large and small infarcts, lacunes, hippocampal sclerosis, cerebral amyloid angiopathy (CAA) and white matter lesions [4]. The cognitive decline that is associated with VaD is believed to be the result of cerebral ischemia secondarily to the vascular changes. Similarly to what is found in AD, amyloid plaques, neurofibrillary pathology, and cholinergic deficits have also been documented in VaD, albeit to a lower degree than what has been found in AD [5].

Behaviorally, patients with VaD show loss in executive functions as an initial symptom, while in patients diagnosed with AD memory loss is often the earliest known symptom [6]. Additional symptoms of VaD include confusion, language deficits, restlessness and agitation, gait disturbances and depression [7]. Risk factors for VaD are predominantly cardiovascular and include, hypertension [8,9], hyperlipidemia [10], atherosclerosis [11], and diabetes [12–14]. Additionally, stroke is an important risk factor for dementia [15,16] with lacunar stroke the most common stroke subtype associated with VaD [17].

Similar to AD, neurofibrillary tangles (NFTs) are a common post-mortem finding in the human VaD brain but are usually present in lower numbers than in AD [5]. In AD, NFTs are composed of hyperphosphorylated forms of tau that accumulate within the entorhinal cortex and CA1 subfield of the hippocampus [18–20]. Besides hyperphosphorylation, post-translational modifications of tau, including proteolysis have been shown to be an important step in the evolution of NFTs. In this regard, numerous studies now support caspase cleavage of tau as an important mechanism contributing to the evolution of NFTs [21,22]. Thus, caspase activation and the cleavage of tau after Asp^421^ is an early event preceding and possibly contributing to NFT formation [23–26].

To date, whether caspase activation and cleavage of tau occurs in VaD is not known despite the fact that ischemia is a well-known activator of apoptotic pathways and a major pathological finding in VaD [4]. Therefore, the purpose of the current study was to investigate the role of caspase-cleaved tau in post-mortem human VaD brain sections using a well-characterized antibody that detects caspase-cleaved tau truncated at Asp^421^ [24]. Our findings are supportive of a role for the activation of caspase-3 and cleavage of tau in VaD, providing further support for the involvement of this family of proteases in NFT pathology.

## Materials and Methods

### Immunohistochemistry

Autopsy brain tissue from seven neuropathologically confirmed VaD cases were studied. Case demographics are presented in Table 1. Fixed hippocampal tissue sections used in this study were provided by the Institute for Memory Impairments and Neurological Disorders at the University of California, Irvine. Approval from Boise State University Institutional Review Board was not obtained due to the exemption granted that all tissue sections were fixed and received from University of California, Irvine. Brain tissue obtained from University of California, Irvine were anonymized and never identified except by case number. Tissue donors or their next of kin provided informed signed consents to the Institute for Memory Impairments and Neurological Disorders for the use of their tissues in research (IRB 2014–1526). Free-floating 40 μm-thick sections were used for immunohistochemical studies as previously described [27]. For bright-field labeling, sections were washed with 0.1 M Tris-buffered saline (TBS), pH 7.4, and then pretreated with 3% hydrogen peroxide in 10% methanol to block endogenous peroxidase activity. Sections were subsequently washed in TBS with 0.1% Triton X-100 (TBS- A) and then blocked for thirty minutes in TBS-A with 3% bovine serum albumin (TBS-B). Sections were further incubated overnight at room temperature with the TauC3 (mouse monoclonal, 1:100). Following two washes with TBS-A and a wash in TBS-B, sections were incubated in anti-rabbit or mouse biotinylated anti-IgG (1 hour) and then in avidin biotin complex (1 hour) (ABC, Elite Immunoperoxidase, Vector Laboratories, Burlingame, CA, USA). The primary antibody was visualized using brown DAB substrate (Vector Laboratories). The periodic acid-schiff (PAS) staining system was purchased from Sigma-Aldrich (St. Louis, MO) and was employed according to the manufacturer’s instruction.

**Table 1:** Case Demographics. PMI, postmortem interval in hours; NPD, neuropathological diagnosis, ND, not determined.

Case	Age	Sex	PMI	NPD	Braak and Braak	Plaque Stage
1	83	M	3.75	VaD	Stage 1	Stage A
2	75	F	10.5	VaD	Stage 2	None
3	83	M	12.4	VaD	Stage 3	None
4	74	M	2.6	VaD	Stage 2	Stage A
5	73	M	4.3	VaD	Stage 0	None
6	88	M	9.9	VaD	ND	Stage A
7	85	F	3.4	VaD	Stage 3	Stage B
8	75	F	2.75	Normal	Stage 2	None
9	78	M	6.00	Normal	ND	None
10	92	F	4.25	Normal	Stage 0	None
11	69	M	6.60	Normal	Stage 0	None

### Immunofluorescence Microscopy

Primary antibodies utilized included the caspase-3-cleaved antibody (rabbit polyclonal, 1:50), PHF-1 (mouse monoclonal, 1:1,000), anti-Aβ (clone 6E10) antibody (mouse, 1:400) and TauC3 (mouse monoclonal, 1:100). The TauC3 antibody was purchased from EMD Millipore (Billerica, MA), while PHF-1 was a generous gift from Dr. Peter Davies (Albert Einstein College of Medicine, Bronx, NY). The anti-Aβ mAb 1560 (clone 6E10) was purchased from Covance (Dedham, MA). The cleaved caspase-3 (Asp175) antibody was purchased from Cell Signaling (Danvers, MA). The AT8, Tau antibody (HT7) and ubiquitin monoclonal antibodies were purchased from Pierce, ThermoFisher Scientific Inc. (Waltham, MA). With the exception of anti-Aβ mAb 1560 (see below), no antigen retrieval methods were employed. For double-label immunofluorescence co-localization studies, experiments were initiated by incubating in primary antibody overnight followed by application of the ABC, Elite Immunoperoxidase kit on day 2 (Vector Laboratories, Burlingame, CA, USA). In this case, instead of completing the staining use DAB substrate, we employed Alex fluor 488-labeled tyramide (green, Ex/Em = 495/519) that was purchased as part of the TSA kit #12 (Life technologies, Green Island, NY). Following labeling with the primary antibody, sections were washed 3X in Tris buffer followed by incubations in Tris A (15 minutes) and Tris B (30 minutes). Sections were then incubated with the second primary antibody overnight at room temperature. On day 3, sections were incubated with secondary biotinylated-SP (long spacer) AffiniPure goat anti-mouse or rabbit IgG for 1 hour (Jackson Immuno Research Labs (West Grove, PA). This was followed by incubation in streptavidin Alex Fluor 555 conjugate for 1 hour (Life technologies, Green Island, NY). Following 3X washes in Tris buffer, sections were mounted and cover slipped using ProLong Gold Antifade with DAPI (Life technologies). To determine if cross-reactivity to reagents was a factor in double-labeling experiments, experiments were replicated with the antibodies in reverse. To visualize beta-amyloid staining, sections were pretreated for 5 minutes in 95% formic acid. To assess apoptosis, the Apoptag peroxidase kit was employed according to the manufacturer’s instructions (EMD Millipore, Billerica, MA).

An Olympus BX60 microscope with fluorescence capability equipped with a MagnaFire SP software system for photomicrography was employed for microscopic observation and photomicrography of the DAB-labeled and fluorescent sections. The fluorescent molecules were excited with a 100-W mercury lamp. Fluorescent-labeled molecules were detected using a filter set having a 460–500-nm wavelength band pass excitation filter, a 505-nm dichroic beam splitter, and a 510–560-nm band pass emission filter.

### Confocal microscopy

For confocal immunofluorescence imaging, the primary antibodies were visualized with secondary antibodies tagged with either Alexa Fluor 488 or Alexa Fluor 555 (Invitrogen, Carlsbad, CA). Images were taken with a Zeiss LSM 510 Meta system combined with the Zeiss Axiovert Observer Z1 inverted microscope and ZEN 2009 imaging software (Carl Zeiss, Inc., Thornwood, NY). Confocal Z-stack and single plane images were acquired with an Argon (488 nm) and a HeNe (543 nm) laser source. Z-stacks images were acquired using a 20x Plan-Apochromat (NA 0.8) objective, emission band passes of 505–550 nm for the detection of the TauC3 (green channel, Alexa Fluor 488) and 550–600 nm for detection of PHF-1 (red channel, Alexa Fluor 555). All images displayed are 2-D, maximal intensity projections generated acquired Z-stacks. Single plane images were acquired with a 63x Plan-Apochromat oil-immersion objective (NA 1.4) with emission long pass of 505 nm for the detection of the TauC3 antibody (green channel, Alexa Fluor 488) and 550–600 nm for the detection of PHF-1 (red channel, Alexa Fluor 555).

### Western blot analysis

Frozen tissue from either frontal cortex or cerebellum was homogenized in TPER buffer (ThermoFisher) and centrifuged (18,000 x g, 10 min). The soluble fraction was removed and protein concentration was determined by the BCA method (Pierce). For each sample, 3 μg of protein were separated by SDS-PAGE (TGX gels, BIO-RAD), transferred to nitrocellulose, and probed with a monoclonal antibody to caspase-cleaved tau.

### Statistical analysis

To determine the percent co-localization, a quantitative analysis was performed as described previously [27] by taking 20X immunofluorescence, overlapping images from three different fields in area CA1 in four separate VaD cases. Capturing was accomplished by using a 2.5x photo eyepiece, and a Sony high resolution CCD video camera (XC-77). For example, to determine the percent co-localization between TauC3 and PHF-1, photographs were analyzed by counting the number of TauC3, PHF-1-positive NFTs alone per 20X field for each case, and the number of cells labeled with both PHF-1 and TauC3. Data are representative of the average number (±S.D.) of each antibody alone or co-localized with both antibodies in each 20X field (3 fields total for 4 different cases). Statistical differences in this study were determined using Student’s two-tailed T-test employing Microsoft Office Excel. To determine any possible correlations between the various groups, Pearson’s coefficients were determined using Microsoft Office Excel.

## Results

### Caspase-cleaved tau immunoreactive pathology

**Fig 1:**
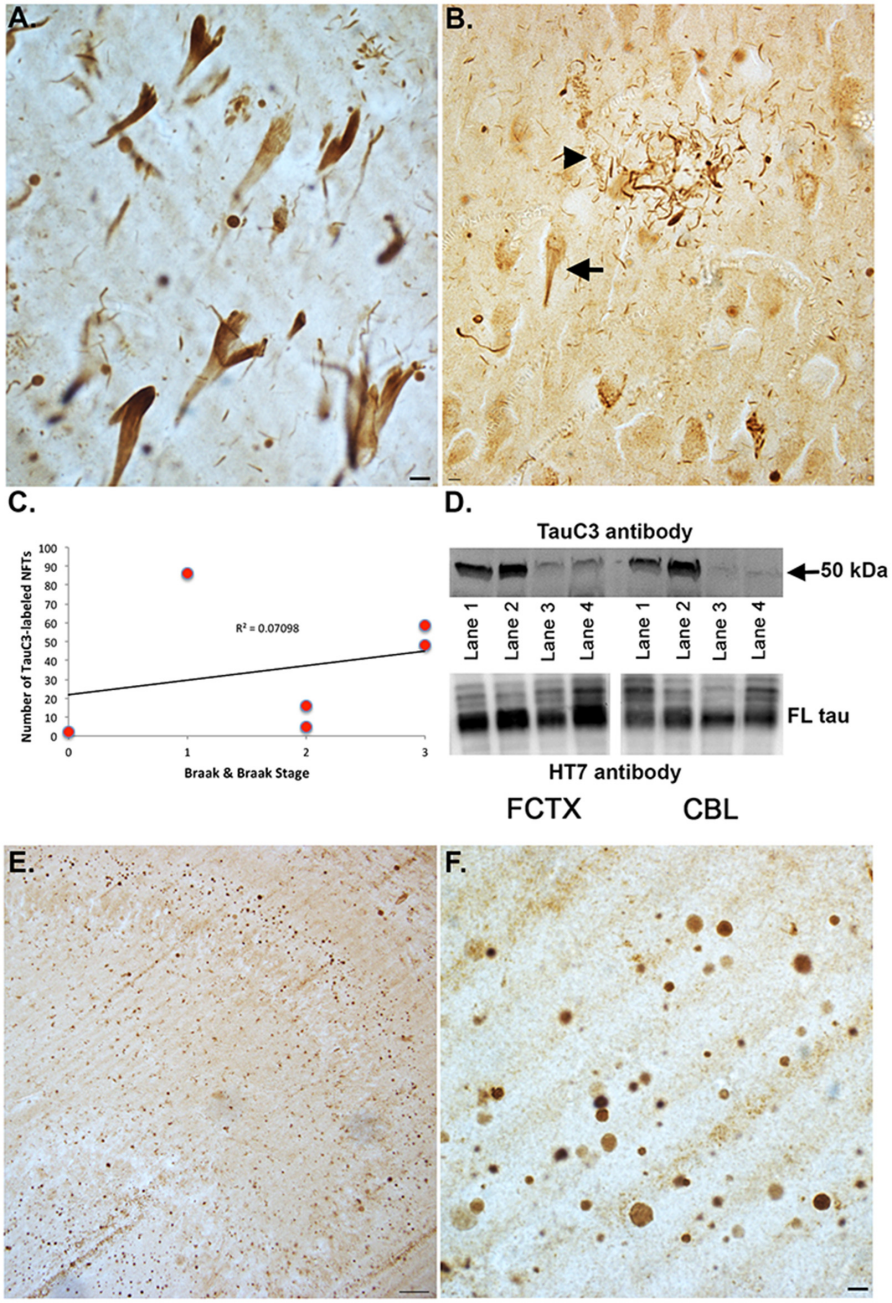
Caspase-cleaved tau in the human vascular dementia brain. **(A)**: Representative labeling from a VaD case utilizing the TauC3 antibody illustrating staining in the hippocampus within apparent NFTs. **(B):** Representative staining in the hippocampus indicating labeling of TauC3 within neuritic plaques (arrowhead), as well as apparent NFTs (arrow). **(C):** Correlation of TauC3 labeling with Braak & Braak stage. For six VaD cases in which the Braak & Braak stage was known, the number of TauC3-positive NFTs was counted three separate times, averaged and then plotted versus Braak & Braak stage. A positive correlation (R^2^ = .070) was observed between these two variables. **(D)**: Western blot analysis utilizing the TauC3 antibody was carried out utilizing brain extracts from the frontal cortex (FCTX) or cerebellum (CBL) of four VaD cases. Lanes 1 (Case 6, Table 1) and 2 (Case 4, Table 1) are VaD cases that had Stage A plaque load, whereas lanes 3 (Case 3, Table 1) and 4 (Case 2, Table 1) were designated as having a plaque load of 0. A band at 50 kDa corresponding to caspase-cleaved tau truncated at Asp^421^ was identified in the FCTX of all four VaD cases and two of four cases in the CBL. The bottom panel of D depicts an identical experiment except transferred proteins were probed with HT7 (1:1,000), an antibody that detects total, full-length (FL) tau. **(E and F):** Low (E) and high magnification (F) of representative labeling from a VaD case utilizing the TauC3 antibody illustrating staining in the dentate gyrus of the hippocampus within numerous, round translucent structures. All scale bars represent 10 μm, except for Panel E, which represents 50 μm.

To determine if caspase-cleavage of tau can be detected in VaD, an immunohistochemical study utilizing the TauC3 antibody was performed utilizing fixed hippocampal brain sections from seven VaD cases. Case demographics for the VaD cases used in this study are presented in Table 1. As an initial step, we screened all seven cases for TauC3 immunoreactivity using bright-field microscopy. The TauC3 antibody reacts with caspase cleaved tau truncated at Asp^421^ [24]. This antibody shows no reactivity with full-length tau or other tau C-terminal truncations and is specific for NFTs, and caspase-cleaved tau within neuritic plaques and neuropil threads [28]. Representative staining is depicted in Fig 1 indicating consistent labeling of TauC3 within NFTs (Fig 1A and 1B, arrow) as well as within neuritic plaques (Fig 1B, arrowhead) of the VaD brain. To determine any possible correlation of TauC3 labeling with NFTs, we quantified the number of TauC3-positive tangles in 6/7 VaD cases in which the Braak & Braak stage was known (Table 1). The results indicated a positive correlation between these two variables (R^2^ = .070) (Fig 1C).

To confirm biochemically that the TauC3 antibody can detect caspase-cleaved tau truncated at Asp^421^, Western blot analysis was performed. In this case we compared two different areas, frontal cortex and cerebellum utilizing four different VaD cases. As shown in Fig 1, a band was observed in all four cases, however, the intensity of the bands appeared stronger in frontal cortex extracts as compared to cerebellum. Because beta-amyloid is thought to be a key initiator in the activation of apoptotic pathways leading to the caspase-cleavage of tau [22], we also compared two cases that pathologically were determined to have a significant beta-amyloid load (Stage A) versus two cases that had minimal beta-amyloid deposition (See Table 1). In this case the band corresponding to caspase-cleaved tau was more robust in those VaD cases with greater beta-amyloid loads (compare lanes 1 and 2 versus 3 and 4, Fig 1D, top panel). As a control, samples were also blotted with HT7, an antibody that detects full-length tau. In this case, total tau appeared to be consistently expressed in each brain region (Fig 1D, bottom panel).

In addition to the labeling of NFTs, application of the TauC3 antibody also revealed staining of numerous round translucent structures (Fig 1E and 1F) within the dentate gyrus of the hippocampus. In this regard, strong immunolabeling with the TauC3 antibody was observed in all seven cases.

### Identification of apparent corpora amylacea in VaD

**Fig 2:**
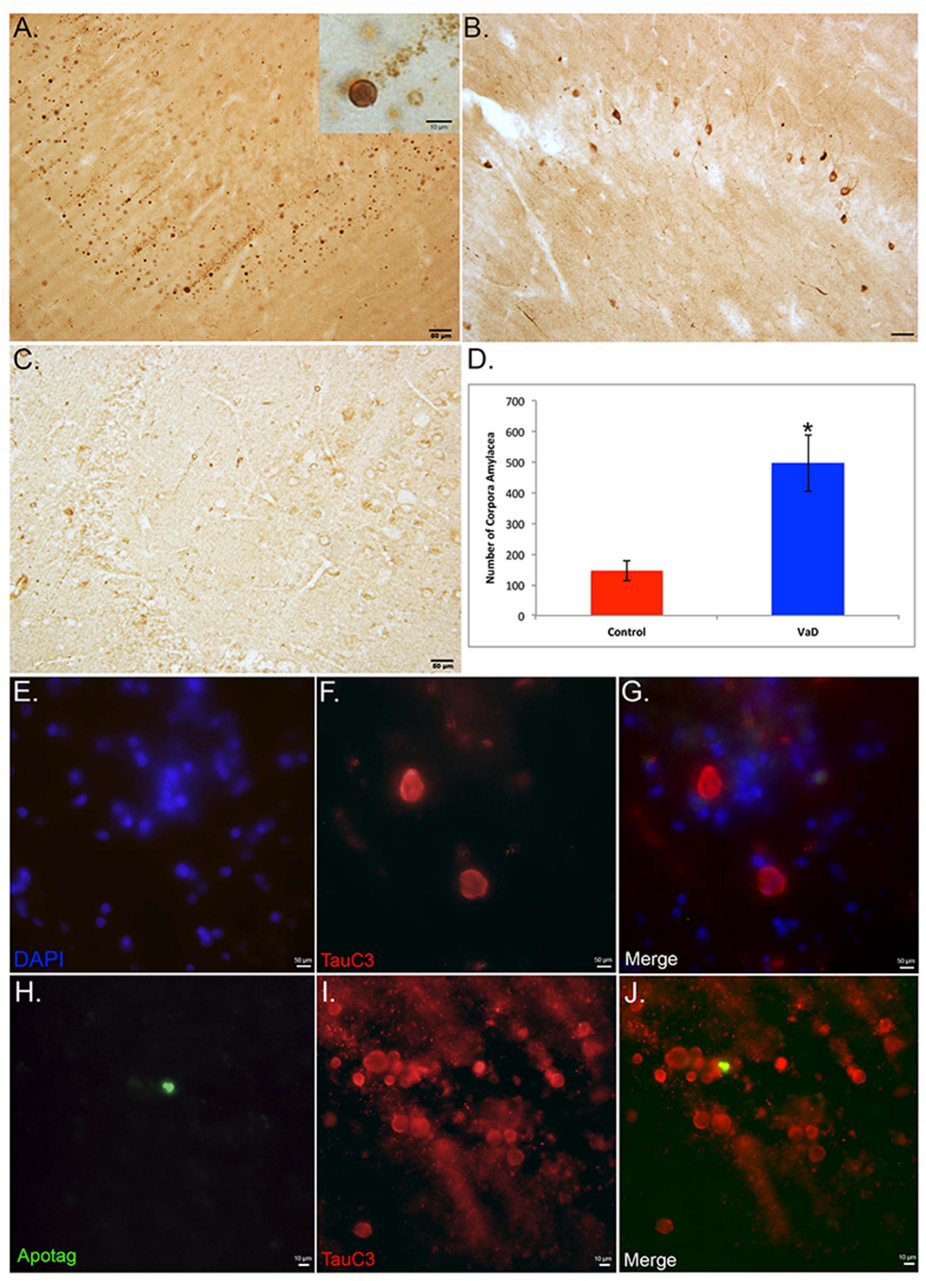
Identification of TauC3-labeled structures as apparent corpora amylacea in VaD. **(A):** Bright-field staining utilizing the TauC3 antibody in the dentate gyrus of a representative VaD showing the presence of numerous round labeled structures that were ring-like in appearance (inset). **(B):** Representative bright-field staining utilizing HT7, an anti-body to full-length Tau did not label these round structures although numerous neurons were labeled. **(C):** Representative labeling of the TauC3 in an aged-matched control case indicating a relative paucity of labeling. Scale bars in Panels A-C represent 50 μm. **(D):** Quantitative analysis of the number of round structures in the hippocampi indicated a significant difference between VaD cases (n = 7, ±S.D.) and aged-matched controls (n = 4, ±S.D.), *p = .008. **(E-G):** Immunofluorescence double labeling in a representative VaD case utilizing TauC3 (red) and the nuclear stain, DAPI (blue) indicated that the round circular structures labeled by TauC3 are not nuclei (merge, G). **(H-J):** Immunofluorescence double labeling in a representative VaD case utilizing TauC3 (red) and TUNEL to label apoptotic cells (green) indicated that the round circular structures labeled by TauC3 are not apoptotic cells by in large (merge, J). Scale bars in Panels E-J represent 10 μm.

Bright-field staining utilizing the TauC3 antibody consistently labeled the presence of numerous round structures that were ring-like in appearance in the dentate gyrus (Fig 2A). To determine if labeling within these structures was specific to caspase-cleaved tau, similar experiments were performed utilizing the anti-tau antibody HT7. Although this antibody labeled numerous neurons in the dentate gyrus region of VaD cases, there was a complete lack of staining within these round structures (Fig 2B). In addition, in age-matched control cases, these structures were only infrequently observed following application of the TauC3 antibody (Fig 2C). Quantitative analysis of these structures in the hippocampus revealed a statistically significant difference in the number of these structures between VaD and age matched controls (Fig 2D). In an attempt to identify these structures, immunofluorescence double labeling was undertaken. Initially double labeling was performed with the TauC3 antibody and the nuclear stain DAPI. Co-localization was not observed (Fig 2E–2G), providing evidence that the spherical structures were not nuclei. To determine if labeled TauC3 structures were apoptotic cells, double labeling was assessed together with Terminal deoxynucleotidyl transferase dUTP nick end labeling (TUNEL). As indicated in Fig 2H–2J, co-localization was not observed providing evidence that these found structures are not apoptotic structures. Based on the morphological appearance of these spherical, translucent structures, we hypothesize they represent corpora amylacea (CA).

### Confirmation of TauC3 labeling within corpora amylacea

**Fig 3:**
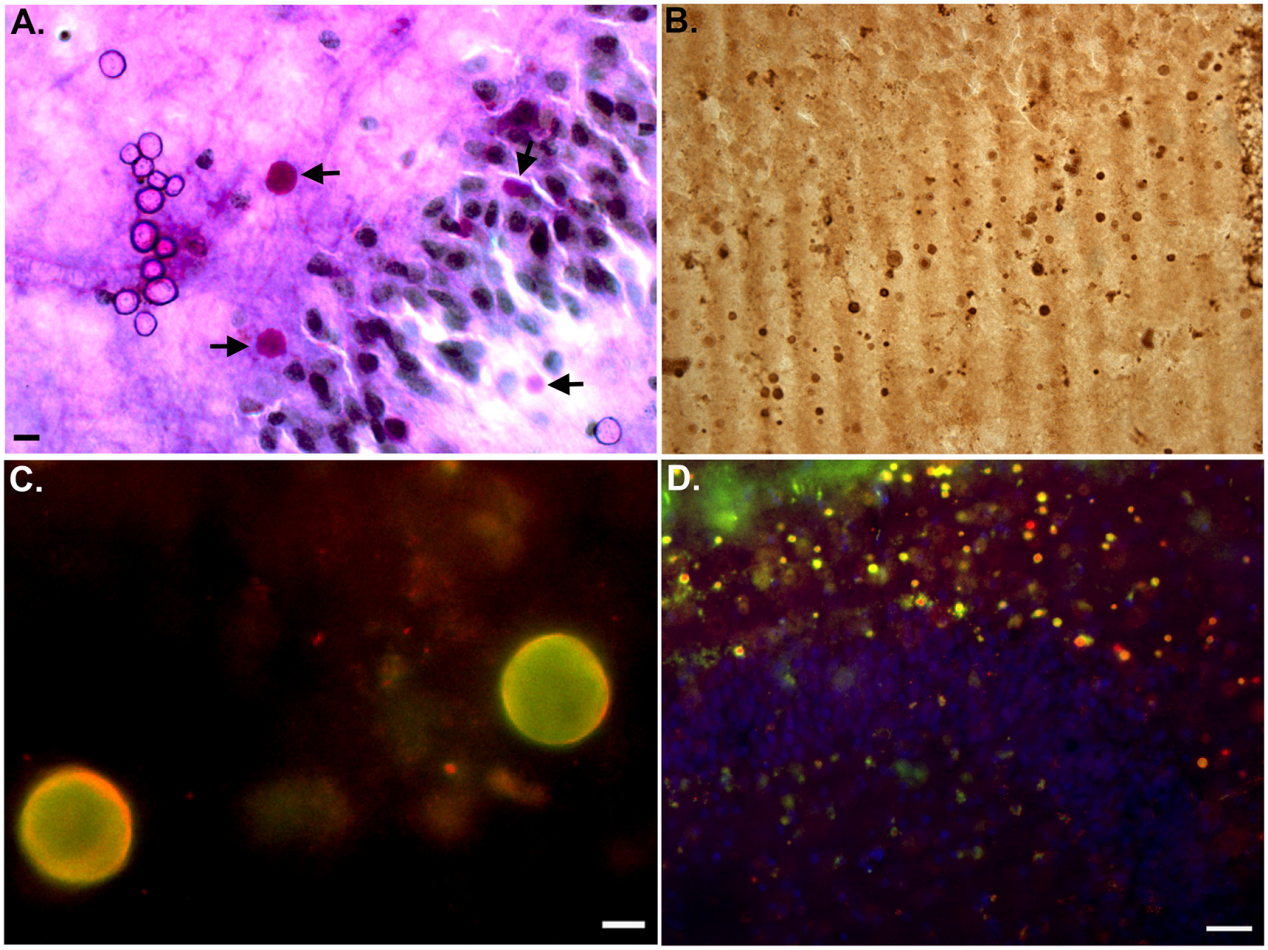
Confirmation of caspase-cleaved tau within corpora amylacea. **(A):** Representative bright field staining in a VaD hippocampal brain section utilizing PAS that specifically labels CA in brain tissue. Labeled CA (magenta color, arrows) were in the vicinity of neurons in the granule cell layer of the dentate gyrus that were counter-stained with hematoxylin. **(B):** Representative bright-field labeling of numerous CA in the hippocampus of a VaD case utilizing an anti-ubiquitin, a specific marker for CA. **(C and D):** Representative immunofluorescence double-labeling in a VaD case at high magnification (C) and low magnification (D) indicating the co-localization of ubiquitin (green) together with the TauC3 antibody (red) within CA. In Panel D, the nuclei were also stained with nuclear stain, DAPI. All scale bars are equivalent to 10 μm except for Panel D, which represents 50 μm.

To confirm these TauC3-positive structures were CA, we stained sections with PAS, a well known specific marker for CA [29]. Labeling of CA with PAS was evident within the same region, the dentate gyrus as for what we observed with TauC3 (arrows, Fig 3A). Additional experiments were undertaken to assess whether these structures stained positive for ubiquitin, another known marker for CA [30]. Application of an anti-ubiquitin antibody revealed an identical staining pattern as compared to TauC3 (Fig 3B) and this same anti-ubiquitin antibody strongly co-localized with TauC3 following double-label immunofluorescence studies (Fig 3C and 3D). Taken together, Figs 2 and 3 supported the presence of truncated tau within CA of the human VaD brain.

### Co-localization of caspase-cleaved tau within NFTs

**Fig 4:**
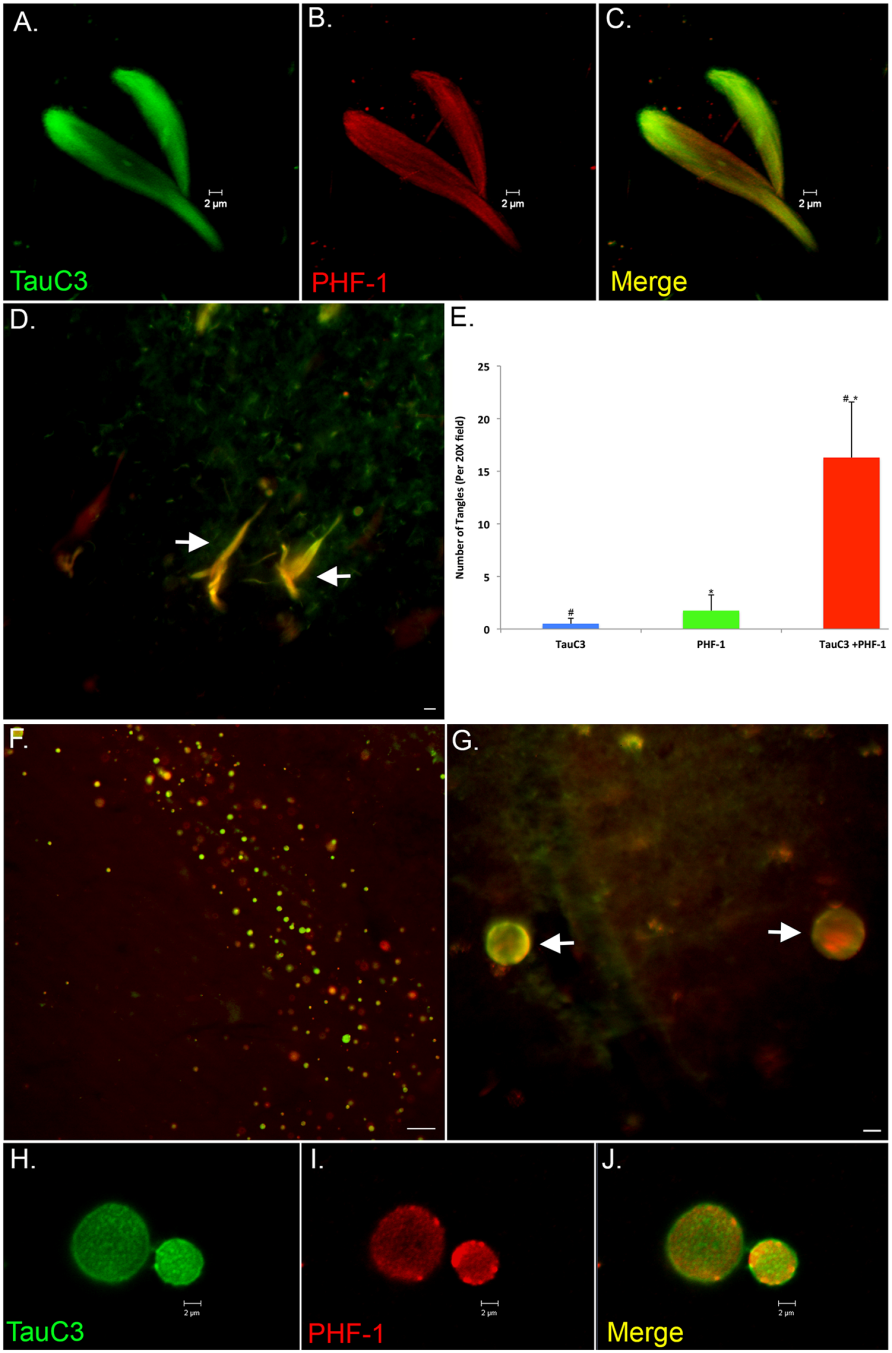
Co-localization of caspase-cleaved tau with NFTs in the VaD brain. **(A-C)**: Representative images from confocal immunofluorescence analysis in VaD utilizing TauC3 (green, A) and PHF-1 (red, B) with the overlap image shown in Panel C. Notice the filamentous nature of staining of PHF-1 as compared to TauC3. **(D and E)**: Representative immunofluorescence double labeling (arrows, D) and quantification of NFTs (E) double-labeled with TauC3 and PHF-1. Data show the number of NFTs labeled with TauC3 alone (blue bar), PHF-1 alone (green bar) or NFTs that were labeled with both antibodies (red bar). NFTs were identified in a 20X field within hippocampi sections by immunofluorescence overlap microscopy (n = 3 fields for 4 different VaD cases) ±S.E.M. *p = 5.46 x 10^-7^ between PHF-1 alone and TauC3 + PHF-1 and #p = 4.49 x 10^-7^ between TauC3 alone and TauC3 + PHF-1. Data indicated that roughly 90% of all labeled NFTS co-localized with both antibodies. **(F and G)**: Low- (F) and High-field (arrows, G) double immunofluorescence overlap images of corpora amylacea within the dentate gyrus of a representative VaD case showing co-localization of TauC3 (green) and PHF-1 (red). **(H-J)**: High magnification confocal images representing labeling of corpora amylacea with TauC3 (H), PHF-1 (I), and the merged image (J). Scale bars represent 10 μm in Panels D and G and 50 μm for Panel F.

To determine the extent of co-localization of caspase-cleaved tau within NFTs, double-labeling immunofluorescence experiments were carried out using PHF-1 as a general marker for NFTs. Confocal analysis revealed strong co-localization with PHF-1 and TauC3 (Fig 4A–4C). A quantitative analysis indicated that approximately 90% of all identified PHF-1 labeled NFTs also labeled with TauC3 (Fig 4E). Strong co-localization of PHF-1 with TauC3 was also observed within CA located within the hippocampus proper of VaD cases (Fig 4F–4J).

### Single-labeling of VaD cases revealed labeling of CA in close proximity to NFTs

**Fig 5:**
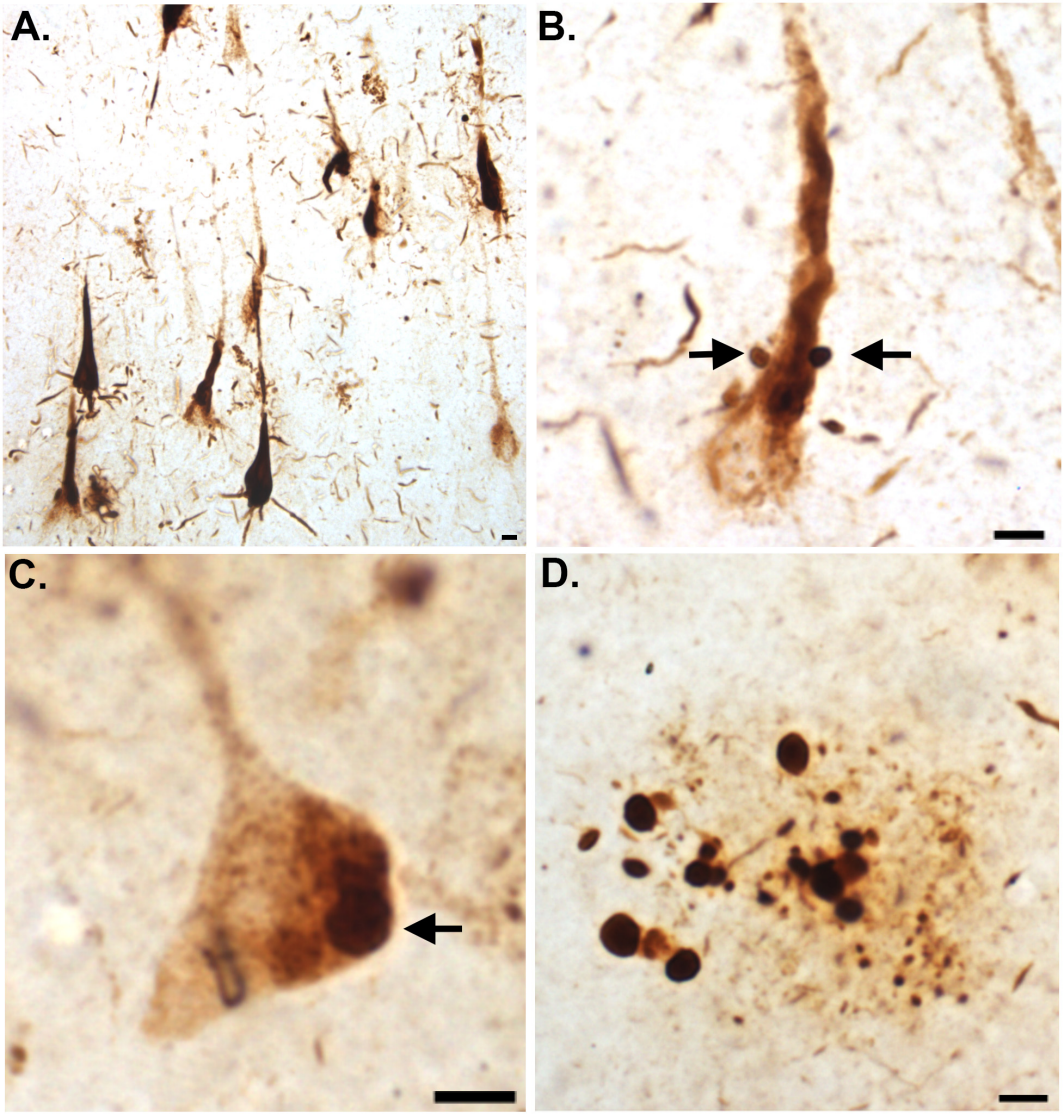
PHF-1 labeling of corpora amylacea within the hippocampus of the VaD brain. **(A)**: Representative bright-field DAB labeling from a VaD case utilizing the PHF-1 antibody illustrating staining in the hippocampus within NFTs. **(B)**: High magnification of a single PHF-labeled NFT indicated the presence of small circular structures (arrows) of the same size and shape as CA in close proximity to the labeled neuron. **(C)**: In this example, a PHF-labeled neuron appeared to exhibit intracellular inclusions that were of the same size and shape as identified CA (arrow). **(D)**: Numerous CA labeled with PHF-1 were documented in plaque-rich regions within the hippocampal region of VaD cases. All scale bars represent 10 μm.

Experiments were also performed using only PHF-1 and bright-field microscopy. As shown in Fig 5, single label immunohistochemical experiments with PHF-1 revealed typical labeling of NFTs throughout the hippocampus (Fig 5A). In a subset of NFTs visualized at high magnification, we noticed the appearance of circular structures of roughly the same size and shape as CA in close proximity to PHF-1-labeled NFTs (arrows, B). That CA may be derived from a neuronal source and represent intracellular inclusions was supported by the presence of labeled structures of the same size and shape as CA within PHF-1 labeled neurons (arrow, Fig 5C). In addition, we found numerous PHF-1-lableled CA within plaque-rich regions in the hippocampus of the VaD brain (Fig 5D).

### TauC3 co-localizes with early tangle markers in the VaD brain

**Fig 6:**
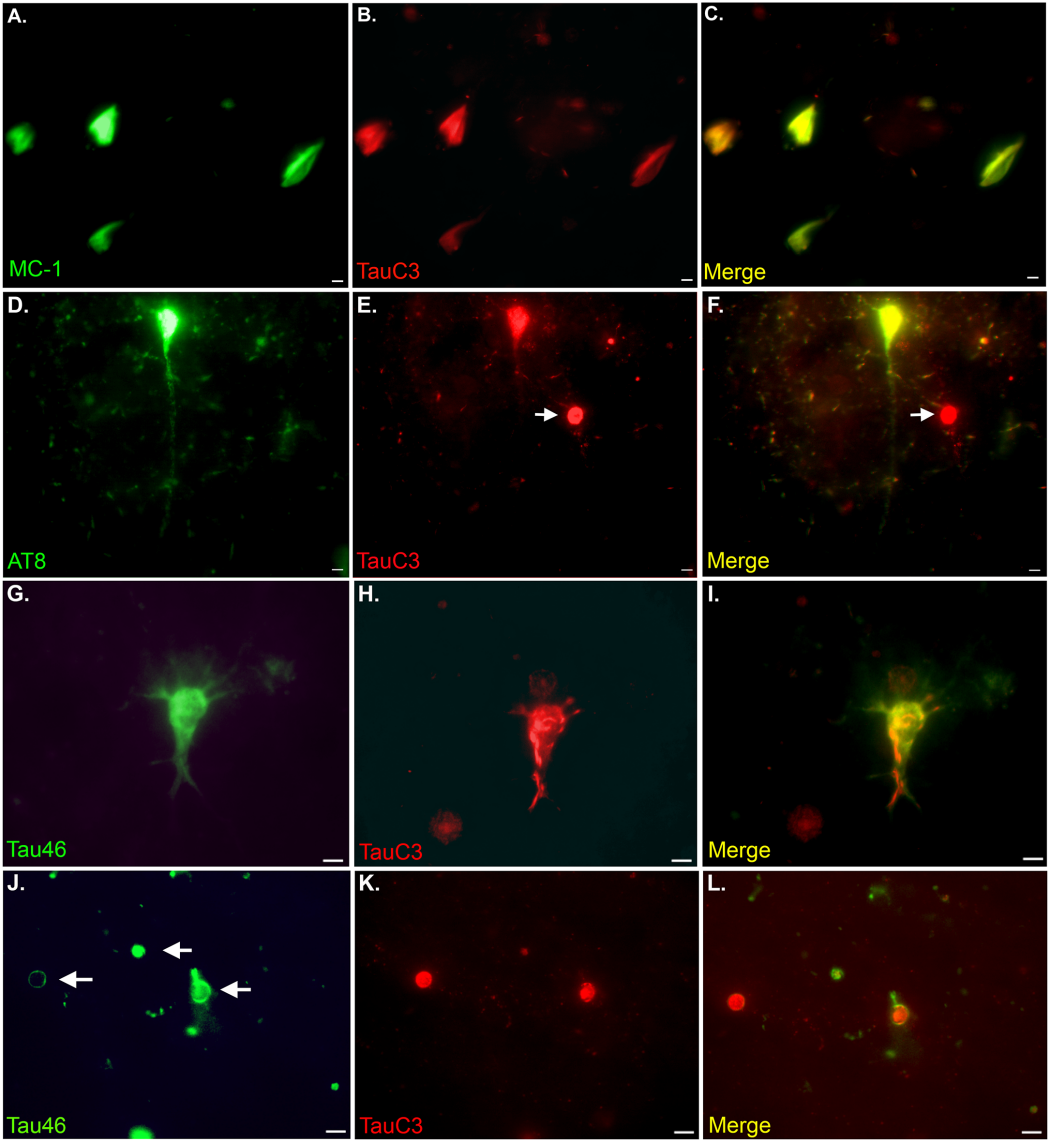
TauC3 co-localizes with early tangle markers in the VaD brain. **(A-C):** Representative images from double-label immunofluorescence analysis in VaD utilizing the early pathological tau marker MC-1 (green, A) and TauC3 (red, B) with the overlap image shown in Panel C indicating co-localization of the two markers (yellow). **(D-F)**: Representative double-label immunofluorescence experiment utilizing the early tangle phosphorylated marker, AT8 (green, D) and TauC3 (red, E) with the overlap image shown in Panel F. Strong co-localization between the two antibodies was observed in tangles, however, CA (arrow, E and F) labeled with TauC3 only. **(G-I):** Identical to Panels A-C except double-label was accomplished utilizing Tau46 (green, G), a C-terminal antibody to full-length tau. Note the distinct subcellular and fibrillary nature of the TauC3 antibody within the labeled neuron (H and I). **(J-L)**: Identical to Panels G-I except the panels depict the labeling by Tau46 of CA (arrows, J) that were also labeled with TauC3 (K and L). All scale bars represent 10 μm.

Previous studies in AD have indicated that the C-terminal truncation of tau is an early event that may facilitate NFT formation [23,24]. Therefore, to examine a similar possible relationship in VaD, co-localization experiments were performed using MC-1 and AT8. MC-1 is a conformational specific antibody that recognizes aberrant folded conformational changes in tau, one of the earliest tau pathological events [31,32]. The antibody AT8 recognizes tau phosphorylated at both serine 202 and threonine 205, which are the first residues to be hyperphosphorylated [33,34]. PHF-1, in contrast, recognizes phosphorylation at serines 396 and 404 and reacts with more mature hyperphosphorylated forms of tau found primarily within late-stage tangles [35]. As shown in Fig 6, double-label immunofluorescence studies utilizing either MC-1 (Fig 6A–6C) or AT8 (Fig 6D–6F) led to strong co-localization with the TauC3 antibody. To assess the relationship between caspase-cleaved tau and full-length tau pathology, fluorescent double labeling for TauC3 and the C-terminal-specific antibody Tau46 [36] was performed. The results revealed a difference in subcellular localization between these two markers, suggesting that both full-length tau (Tau46, green) and cleaved tau (TauC3, red) are present within the same NFTs (Fig 6G–6I). Because the C-terminal epitope recognized by Tau46 has been shown to be liberated by executioner caspases [23], these results confirm the specificity of the TauC3 antibody for the C-terminal cleavage site within tau.

It is noteworthy that although neither MC-1 nor AT8, labeled CA, application of the T46 antibody immunolabeled a subset of CA that co-localized with the TauC3 antibody (Fig 6J–6L). Representative staining of additional VaD cases with the antibodies in reverse order gave similar results (S1 Fig).

### Caspase-cleaved tau in neuropil threads within plaque-rich regions

**Fig 7:**
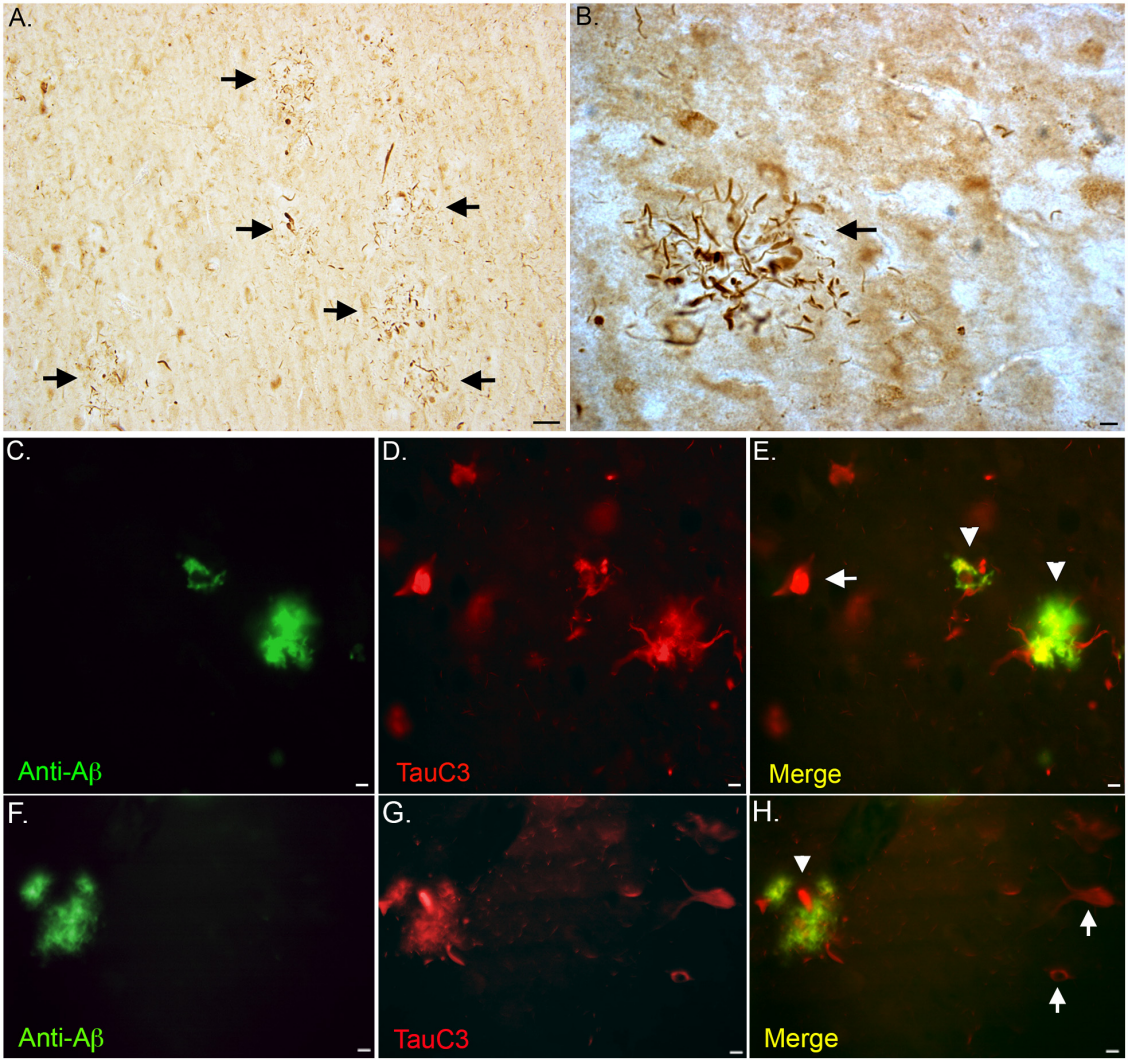
Caspase-cleaved tau within neuritic plaques in the VaD brain. **(A and B)**: Representative bright-field microscopy showing TauC3 labeling of neuritic plaques at low-field (arrows, A) and at high magnification (arrow, B). **(C-E)**: Representative immunofluorescence double labeling utilizing an anti-Aβ (clone 6E10) antibody (green, C), TauC3 (red, B), and the merged image (C) indicating co-localization of two markers (yellow, arrowheads). The arrow in Panel E reflects the labeling of a single NFT by TauC3 that is not labeled with the 6E10 antibody. **(F-G)**: Identical to Panels C-E depicting labeling in an additional VaD case. All scale bars represent 10 μm except Panel A, which represents 50 μm.

In addition to labeling CA and NFTs, the TauC3 antibody appeared to label neuropil threads within plaque rich regions in VaD cases (Fig 7A and 7B). To confirm the presence of caspase-cleaved tau within neuropil threads of extracellular plaques, immunofluorescence double labeling was performed with the anti-Aβ (clone 6E10) antibody. As shown in Fig 7C–7E co localization between TauC3 and 6E10 was evident within extracellular plaques. Unlike labeling within NFTs, we did not observe consistent labeling of the TauC3 within neuropil threads within plaque-rich regions in all seven VaD cases examined (data not shown). Additional double-labeling with anti-Aβ and TauC3 in another representative VaD case is shown in Fig 7F–7H.

### Caspase-3 activation

**Fig 8:**
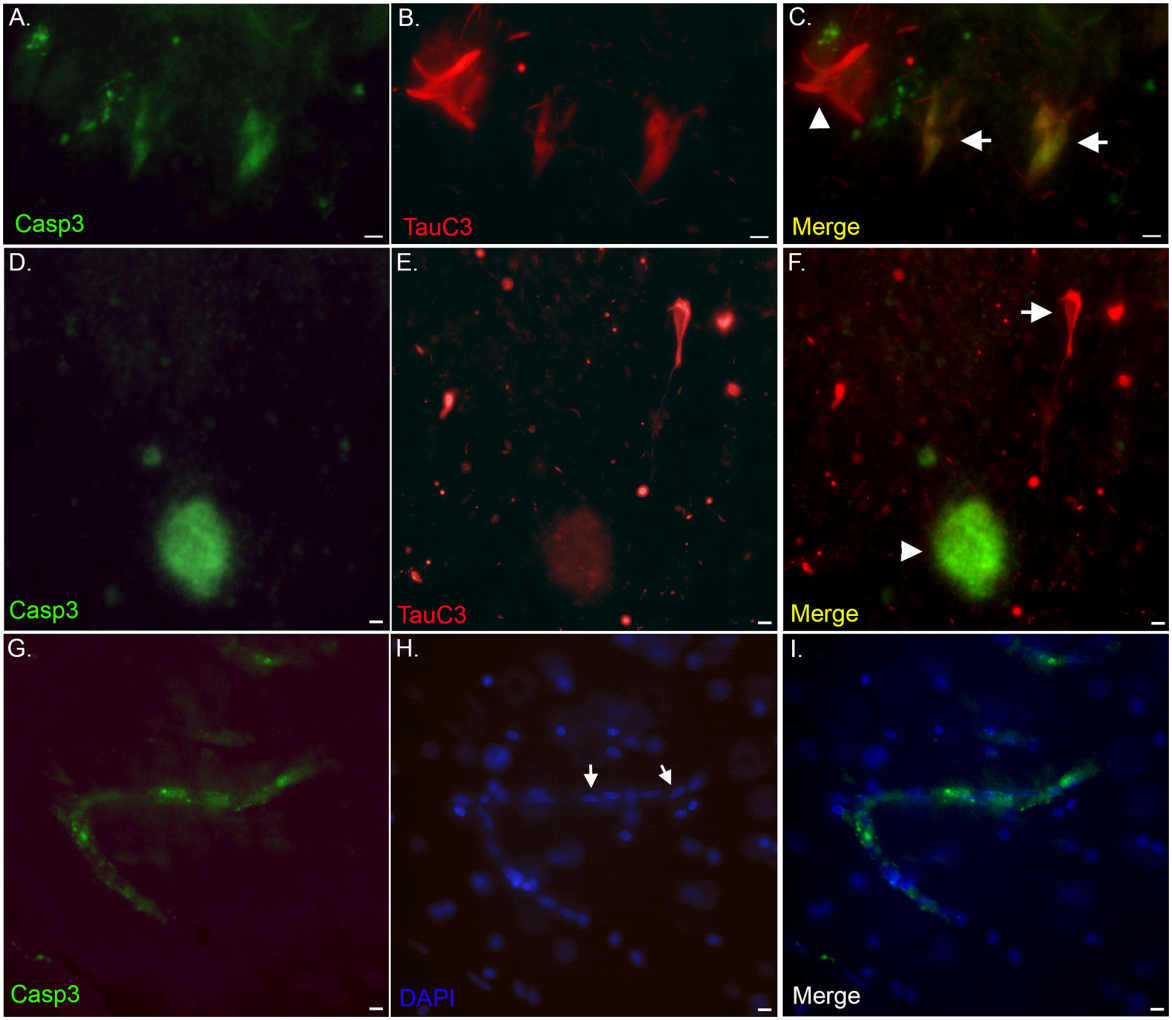
Caspase-3 activation in the VaD brain. **(A-F):** Representative immunofluorescence double labeling within the human VaD brain utilizing an antibody to active caspase-3 (green, Panels A and D) and TauC3 (red, Panels B and E), with the overlap images shown in Panels C and F. Labeling of active caspase-3 was evident within pretangles that co-localized with TauC3 (arrows, C). Co-localization of the two antibodies was also evident within plaques although TauC3 gave a much weaker fluorescence signal (F). In fibrillary NFTs only TauC3 was present (arrowhead, C and arrow, F). **(G-I)**: Representative immunofluorescence double labeling with active caspase-3 (green, G) and the nuclear stain, DAPI (blue, H) indicating labeling within blood vessels of the VaD brain (I). Note the appearance of cuboid, elongated nuclei that typically define endothelial cell nuclei (arrows, H). All scale bars represent 10 μm.

In a final set of experiments we sought to determine whether active caspase-3 co-localizes with TauC3 utilizing an antibody that specifically detects the active fragment of caspase-3 following cleavage at aspartate 175 of the enzyme. We were unable to detect co-localization of the two antibodies within fibrillar NFTs (Fig 8A–8F). However, we were able to detect faint caspase-3 labeling that co-localized with TauC3 within neurons that appeared morphologically to represent pretangles. Pretangles are defined as containing cytoplasmic tau immunoreactivity without apparent formation of fibrillary structures [37]. Activated caspase-3 was also found in plaques and blood vessels of the VaD brain (Fig 8D–8I). It is noteworthy, that labeling of pretangles with the TauC3 antibody was the exception not the rule and in general resulted in a much weaker immunofluorescence signal than TauC3 labeling of mature NFTs. Unlike for TauC3, active caspase-3 labeling was never identified within CA (Fig 8E and 8F).

## Discussion

VaD is the seconding leading cause of dementia in the USA, and has a higher negative predictive value on survival in comparison with patients affected by AD [38]. Specific conditions that increase the potential for strokes or microbleeds including hypertension, hyperlipidemia, and atherosclerosis are important risk factors for VaD. Currently there is a lack of a widely accepted neuropathological criteria for VaD [3]. It has been estimated that 25–80% of all dementia cases show mixed pathologies between VaD and AD making it difficult to diagnose pure VaD [2]. Similarly to what is found in AD, amyloid plaques, neurofibrillary pathology, and cholinergic deficits have also been documented in VaD, albeit to a lower degree then what is observed in AD [5]. Although stroke is a well-known risk factor for VaD [16,17,39], whether the subsequent ischemia and potential activation of caspases occurs in VaD has not been investigated. Therefore, the purpose of the current study was to investigate the potential activation of caspases by examining caspase-cleaved tau in post-mortem human VaD brain sections using a well-characterized antibody (TauC3) that detects caspase-cleaved tau truncated at Asp^421^ [24].

Screening seven pathologically confirmed cases of pure VaD (Table 1) with the TauC3 antibody revealed three consistent staining features: 1) labeling of TauC3 within NFTs; 2) identification of caspase-cleaved tau within apparent corpora amylacea; 3) labeling of neuritic plaques. NFTs are a common post-mortem finding in the human VaD brain but are usually present to a lower degree when compared to AD [5]. In AD, NFTs composed of hyperphosphorylated forms of tau accumulate within the entorhinal cortex and CA1 subfield of the hippocampus [18–20]. In addition to hyperphosphorylation, post-translational modifications of tau, including proteolysis have been shown to be an important step in the formation of NFTs. In this regard, numerous studies now support the caspase cleavage of tau as an important mechanism contributing to the evolution of NFTs [21,22]. Thus, caspase activation and the cleavage of tau after Asp^421^ is an early event preceding and possibly contributing to NFT formation [23–26]. Our findings are supportive of a role for caspase-cleavage of tau in VaD, providing further support for the involvement of this family of proteases in NFT pathology. To corroborate these findings, we performed double-label experiments utilizing an antibody that detects active caspase-3. Although labeling with this antibody was observed in plaques, blood vessels, and pretangle neurons, we did not observe staining within fibrillar NFTs that labeled with PHF-1. These findings suggest that caspase-3 activation precedes caspase-cleavage of tau, and is no longer active in mature tangles, possibly due to turn over of the enzyme that is present in nominal concentrations within neurons. Our findings in VaD are in aligned with what has been observed in AD, namely that caspase activation and cleavage of tau is an early event that contributes to the evolution of NFTs [23–26]. One mechanism that may activate apoptotic pathways in VaD is the presence of beta-amyloid. Previous studies have supported a role for beta-amyloid in initiating the activation of apoptotic pathways leading to caspase-3 activation and the C-terminal cleavage of tau [22–24]. In the present study, we were able to demonstrate by Western blot analysis that caspase-cleaved tau was significantly greater in VaD cases in which beta-amyloid deposition was confirmed post-mortem. These data would support that caspase-cleaved tau links beta-amyloid deposition to NFT formation as has been previously shown in AD [22–24]. Interesting, our Western blot analysis also revealed the presence of caspase-cleaved tau in the cerebellum. This result may not be all that surprising considering that cerebellar dysfunction has been postulated to play an important role in VaD [40]. In a previous immunohistochemical study we demonstrated the presence of caspase-cleaved tau in the cerebellum of the Alzheimer’s disease brain despite the lack of beta-amyloid plaques in this region [41]. In the present study, screening of the cerebellum for beta amyloid by immunohistochemistry did not reveal any deposition of beta-amyloid (data not shown). Therefore, the presence of caspase-cleaved tau in the cerebellum of both the Alzheimer’s and vascular disease brain does not appear to be directly related to the presence of beta-amyloid in this brain region.

In addition to NFTs, the TauC3 antibody consistently labeled numerous translucent round structures in the dentate gyrus of the hippocampus proper. The lack of colocalization between TauC3 and Terminal deoxynucleotidyl transferase dUTP nick end labeling as well as with DAPI within these structures argue against these structures being apoptotic cells or nuclei. Based on the morphological appearance as well as positive labeling with PAS and ubiquitin antibodies we conclude that these structures are corpora amylacea (CA). CA are spherical, laminated, basophilic to eosinophilic structures located in the subpial, periventricular and perivascular regions [42,43]. It is note worthy, that the identified CA in the current study were not found in these regions but instead were prominent in the granule cell layer of the hippocampus. CA are inclusions found to accumulate in the central nervous system and are associated with normal aging as well as neurodegeneration [42]. Reports have shown that approximately 4% of the total weight of CA is composed of protein and that ubiquitin may be one of the primary protein components [30]. The presence of ubiquitin suggests that the accumulation of altered proteins may be involved in the pathogenesis of CA [30]. In addition to ubiquitin, studies have found CA to be reactive with anti-tau and to be present in larger numbers in neurodegenerative disease brains versus that of normal ageing brain [44–46]. Our results revealed the presence of caspase-cleaved tau within CA in the dentate gyrus and the number of labeled CA were significantly higher than what was observed in age-matched controls. Interesting, DAB staining of VaD cases with PHF-1 revealed immunoreactivity in apparent CA that were localized near or within labeled neurons (Fig 5). Our results are suggestive that CA originated as intracellular neuronal inclusions and these findings are supported by previous studies [44,47]. We hypothesize that tau may be modified by post-translational processes that includes phosphorylation and proteolysis and incorporated into these spherical structures. It has been suggested that CA are involved in the sequestration of potentially hazardous products of cellular metabolism including the presence of polymerized proteins [42,48]. Our data showing the presence of caspase-cleaved tau as well as positive staining with PHF-1 would support this notion and suggests that CA may play a protective role similar to what has been ascribed for Hirano bodies [49].

In conclusion, we investigated a potential role for caspase-cleaved tau in VaD utilizing a well-characterized antibody that specifically detects caspase-cleaved tau truncated at Asp^421^. We found that application of TauC3 revealed consistent labeling within NFTs, neuritic plaques, and CA in the human VaD brain. The presence of caspase-cleaved tau within CA that were regionally localized within the dentate gyrus is a novel finding. The localization of CA within the hippocampus proper and not in perivascular regions is suggestive that they may be involved in the disease pathogenesis. However, whether the presence of CA in VaD is contributing factor or simply a product of the disease process is not known and will require further investigation. Staining of the TauC3 antibody co-localized with PHF-1 within the majority of NFTs and our data are suggestive that caspase activation precedes tau cleavage in NFTs. Collectively, these data support a role for the activation of caspase-3 and proteolytic cleavage of TauC3 in VaD providing further support for the involvement of this family of proteases in NFT pathology.

## Supporting Information

S1 Fig TauC3 co-localizes with early tangle markers in the VaD brain. (DOCX)
