# Supplementary material for: Caspase-Cleaved Tau Co-Localizes with Early Tangle Markers in the Human Vascular Dementia Brain
Source: PLoS One. 2015 Jul 10;10(7):e0132637. doi: 10.1371/journal.pone.0132637 (PMC4498690; doi:10.1371/journal.pone.0132637)

**S1 Fig.** **TauC3 co-localizes with early tangle markers in the VaD brain. (A-C):** Representative images from double-label immunofluorescence analysis in VaD utilizing TauC3 (green, A) and the early pathological tau marker MC-1 (red, B) with the overlap image shown in Panel C indicating co-localization of the two markers (yellow). **(D-F)**: Representative double-label immunofluorescence experiment utilizing TauC3 (green, D) and the early tangle phosphorylated marker, AT8 (red, E) with the overlap image shown in Panel F. **(G-I):** Identical to Panels A-C except double-label was accomplished utilizing TauC3 (green, G), together with Tau46, a C-terminal antibody to full-length tau (red, H) with the overlap image shown in Panel I.


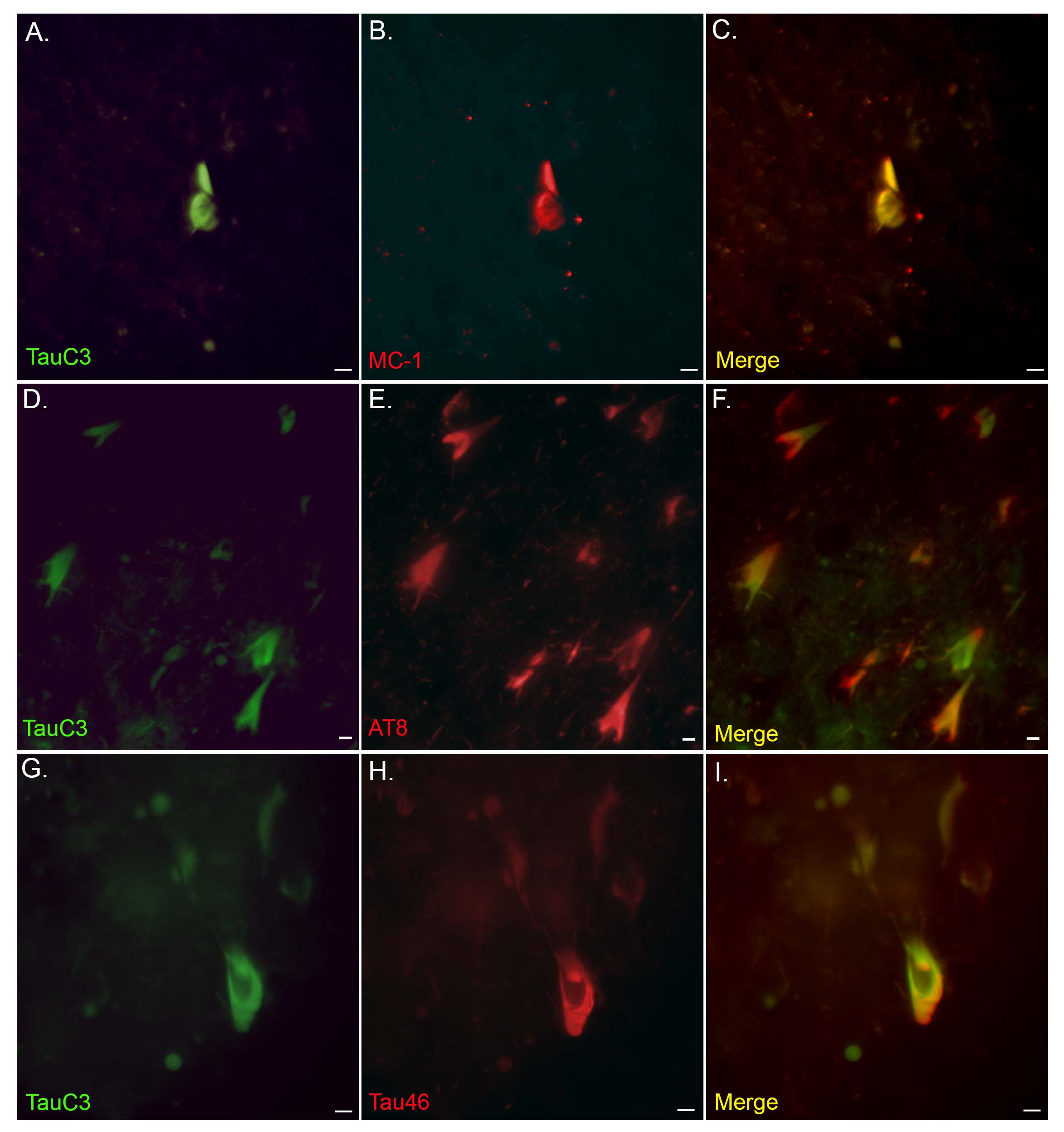

Supplement: S1 Fig — (DOCX) [file pone.0132637.s001.docx]
